# Exploring whether (and how) self-reflection can improve practice as a teacher educator

**DOI:** 10.15694/mep.2018.0000067.1

**Published:** 2018-03-23

**Authors:** Konstantinos C. Fragkos

**Affiliations:** 1 Medical School

**Keywords:** self-reflection, self-study, self-research

## Abstract

This article was migrated. The article was marked as recommended.

The present essay describes a model under which a method of self-improvement of teacher-educator practice can be implemented by analyzing personal practices. I will focus my argument on investigating self-reflection and the subject under investigation will be the educator: how can they self-reflect in order to enhance their learning and hence their practice. The first step of the model is self-reflection. The second step will be self-study. The final step will be self-research techniques (such as autoethnography). This final step will provide the validity for improving personal practice in a possibly reliable way such that practices of narcissism and self-replicating redundancies or errors are avoided. By acknowledging the multiple identities a teacher assumes in their professional practice allows them to analyze them systematically and eventually improve on them.

## Introduction

*One of the problems in teaching is that teachers tend to act before they even have an understanding of how things work or how things have developed ( Koster and van den Berg 2014).*

It is not unreasonable to state that teaching is what an educator’s professional purpose is. It is their daily practice, through which they will pursue students’ learning. Their tools are their formal knowledge, obtained through formal education, but also their experience, which emerges from practicing teaching. Hence, their own practice becomes a means to learn as they progress professionally ( Day *et al.* 2006; Lamote and Engels 2010). Teaching and learning thus become convoluted concepts for an educator who essentially acts both. Schön (1983, 1987) based his epistemology of practice on the educator’s need to learn through their practice. He believed that the existing epistemology in education was not enough to explain professional artistry, which arose through situations, normally not taught about, but learned though professional practice. Thereupon, his theoretical model of reflection was born, which currently permeates all educational levels and holds an important role in adult learning, alongside other important theoreticians ( Fragkos 2016; Mann *et al.* 2009; Norrie *et al.* 2012; Williams 2001).

*I am confused. As a person, I learn. But I also teach in my professional practice. Can I teach and learn at the same time? Maybe yes. Can I teach others how to learn? Maybe yes. Can I teach? Can I learn? Can I teach how to learn? Can I learn how to teach? Can I teach how to teach? Can I learn how to learn?*

*Anonymous teacher*

Under the prism of convoluted teaching and learning, reflecting on an educator’s identity can get disconcerting since the identities of the learner and teacher start to coalesce ( Dinkelman 2011; Izadinia 2014). More so, since an educator likely has a view that they have already learnt and know. Joseph C. Senese (2005) provides some thoughts on this:

*.. I recognize that who I am, not only as a teacher but also as a person, influences my teaching and ultimately my students’ learning. Conversely, because I identify myself as a learner in my high school English classes, I recognize that interactions and relationships with students influence who I am ( Senese 2005).*

Hence, defining teacher identity becomes challenging, seeming all the more like a modern ouroboros: which element comes first, teaching or learning? Gee (2000) models identity under four perspectives:

*..Four interrelated ways of thinking about what and who we are: nature (identity by nature), institutional (identity by the positions we hold), discursive (identity by what we have done and in dialogue with others) and affinity (identity by allegiance to practices and perspectives of group affiliation) ( Dinkelman 2011).*

So, the issues of *self* and reflection are crucial here, influenced by different states of agency, emotion, narrative, and discourse alongside extrinsic factors ( Beauchamp and Thomas 2009; Lamote and Engels 2010). It has been said that the teacher educator teaches but is also learning. I will advocate in the present essay that teacher learning can be achieved with self-reflection of their own practice. I will focus my argument on investigating self-reflection and the subject under investigation will be the educator: how can they self-reflect in order to enhance their learning and hence their practice. I will provide an exemplary context which pertains to this question.

## Contextual example

Doctors in the UK currently have many roles in their professional posts. It will usually be a combination of clinical duties alongside research and educational activities. For the clinical part, this can seemingly involve ward rounds, outpatient clinics, interventional procedures, and multidisciplinary team meetings. Research-wise, duties involve clinical observations, conducting clinical trials, data collection and analysis. Finally, educational activities involve teaching undergraduate medical students in lectures or by-the-bedside, teaching postgraduate students, supervising projects; also clinical supervision of junior doctors.

As part of their practice, reflection is imperative either in training where it is needed with annual review of clinical progress or once fully trained for revalidation and continuing professional development ( Bernard *et al.* 2012; Koole *et al.* 2011; Ng *et al.* 2015; Wald 2015). In their acting, however they do so under multiple identities ( Day *et al.* 2006):

1. Medical trainee and professional identity. This identity has to do with professional conduct and relates to undergraduate education in medicine, postgraduate education in medicine as well as current medical practice.
2. Teacher as educator identity: this relates to how teaching practice is perceived in relation to students, colleagues and all related education practices.
3. Teacher as student identity: this relates to how being a teacher is learnt. This is an identity assumed when being educated as to how to improve practice as an educator.

This essay will focus on the identity as teacher and in particular the identity as teacher who will try to learn through their practice (hence educator and student teacher). Although reflection is very much linked with healthcare education already ( Buckley *et al.* 2009; Chaffey *et al.* 2012; Chen and Forbes 2014; Crowe and O’Malley 2006; Epp 2008; Fragkos 2016; Jayatilleke and Mackie 2013; Kuiper and Pesut 2004; Lethbridge *et al.* 2011; Mann *et al.* 2009; McGillivray *et al.* 2015; Miraglia and Asselin 2015; Ng *et al.* 2015; Nguyen *et al.* 2014; Norrie *et al.* 2012; Prasko *et al.* 2012; Rushmer *et al.* 2004; Tsingos *et al.* 2015a; Tsingos *et al.* 2015b; Van Roy *et al.* 2015; Williams 2001), I will try to focus on self-reflection which will improve personal practice as a teacher educator.

Overall, a model will be suggested under which an efficacious method of self-improvement of teacher-educator practice can be implemented by analysing personal practices. I will suggest the first step is self-reflection; the necessary theory for reflection is given in the first section of the literature review. The second step will be self-study. The necessary theory will also be presented in the literature review. The final step will be self-research techniques (such as autoethnography). This final step will provide the validity for improving personal practice in a possibly reliable way such that practices of narcissism and self-replicating redundancies or errors are avoided. I will conclude the essay with a summary of suggestions. The method for suggesting this model will be a literature review of techniques that enhance self-reflection. This literature review will not be systematic but it will be as comprehensive as possible and will be relevant.

## Self-reflection and reflective practice in healthcare education: how many type of reflections are there?

Reflective practice in healthcare education has been an intriguing topic for quite a few decades now ( Argyris and Schön 1978; Boyd and Fales 1983; Dewey 1933; Glazer 1974; Kolb 1984; Polanyi 1966; Schön 1983; Sosa 2011; Van Manen 1977). Although it ranges and affects the whole domain of education and professional practice, I will focus on its aspects related healthcare education. In healthcare, there is a constant debate of accountability. Accountability towards society, accountability towards patients and accountability towards any possible stakeholder ( Crowe and O’Malley 2006). The history of healthcare sciences is filled with examples where re-evaluation of existing ideas or paradigms was forced by ongoing problematic and problematizing areas. Healthcare education is all the more important in this context ( Fragkos 2016; Frenk *et al.* 2010).

Theories of healthcare education have largely focused on the inadequacies of current education systems which focuses on producing graduates who satisfy pre-determined criteria but might not necessarily believe them, live with them or act upon them. In other words, there has been an observed disparity between learning and then professional practice. Frenk *et al.* (2010) have demonstrated that there is a mismatch between professional competencies and patient and population priorities, resulting from fragmentary, outdated, and static curricula that produce ill-equipped graduates from underfinanced institutions ( Fragkos 2016).

The question of senior educators internationally has been how to ensure learning that will affect genuinely educators’ future actions. Reflection has emerged as one of the ways to achieve this ( Larrivee 2000). Hence, a full range of reflections has stemmed from this notion: reflection during education, reflection during practice, reflection as part of professional progress. For example, appraisals (with reflective interviews with appraisers) have been chosen as the way to ensure ongoing competence for registration as a healthcare professional. This is quite prominent in the UK with bodies such as the General Nursing Council or the General Medical Council requiring annual or every five years appraisals to allow you to register for a license to practice ( Archer and de Bere 2013; Bolsin *et al.* 2015; Dawda 2013; Murphy *et al.* 2012; Wright *et al.* 2016).

The development of reflective theory has generated explanations regarding the development of knowledge and learning process during our daily practice leading reflection to be considered a cornerstone of all established education practices. When one starts to think about reflection, you can easily grasp that it involves thinking because you think about actions or facts that have taken place. However, you quickly understand that this definition is lacking in that reflection also involves changing actions and practices. And then you can easily discern that emotions play an important part. And finally, is it static or dynamic; does it happen as a once-off procedure or does it need repetition? In other words is it a process? Hence, how best to define reflection?

Focusing on the healthcare sciences, a recent umbrella review concluded that the recent definition by Nguyen *et al.* (2014) was concise but very generic as well: they propose a conceptual model for reflection that identified five core components of reflective practice categorised with respect to content (thoughts and actions), process (attentive, critical, exploratory and iterative process), or both (underlying conceptual frame and the view on change and the self). Their analysis is summarised in this definition:

*Reflection is the process of engaging the self in attentive, critical, exploratory, and iterative interactions with one’s thoughts and actions, and their underlying conceptual frame, with a view to changing them and with a view on the change itself ( Nguyen et al. 2014).*

This definition also took into account the trigger and context of reflection as extrinsic elements to complete the reflection model. (e.g. experience and timing). This broad but accurate definition encompasses elements from multiple definitions from theorists of reflective practice (e.g. Dewey (1933); Kolb (1984); Mezirow (1991); Schön ( 1983, 1987)) intensifying the iterative process and the vertical dimension of reflection. For example they curly relate to the iterative scheme by Schön (1983): knowing-in-action; surprise; reflection-in-action; experimentation; and reflection-on-action or the one by Boud *et al.* (1985): returning to experience; attending to feelings; re-evaluation of experience; and outcome/resolution ( Mann *et al.* 2009).

Williams (2001) separates critical reflection from self-reflection in his now famous definition:

*Professional education scholars concur that specialized knowledge is clearly essential for professional practice; however, they also suggest that self-consciousness (reflection) and continual self-critique (critical reflection) are crucial to continued competence. [..] Reflection is an examination of the content or description of an issue or problem and involves checking on the problem solving strategies that are being used-[..] an examination of ‘What?’ and ‘How?’. [..] Critical reflection is stimulated by perceived discrepancies between a learner’s beliefs, values, or assumptions and new information, knowledge, understanding, or insight, [..] a dialogue journal which describes the learner’s self-analysis and the educator’s or fellow learner’s responses is one strategy for stimulating critical reflection.*

Based on the ambitious theoretical underpinnings by Donald Schön, John Dewy, Jack Mezirow and others, many practitioner sand researchers have sought out to apply and quantify reflective activities. In doing so, an epistemological paradox has emerged: reflection appears to be influenced more by a reductionist approach aligned with dominant epistemological positions in medicine, such as evidence-based medicine, than by the historically critical (artistic) philosophical underpinnings ( Fragkos 2016; Koole *et al.* 2011; Ng *et al.* 2015). This problematic area is currently under discourse and will not be explored in depth in the present essay.

Based on these models and theories, various techniques for reflection have been tested in healthcare education during design and evaluation. Summative reflective technique methods include portfolios (paper or electronic), reflective diaries/autobiographical stories (paper or electronic), critical incident reports/essays and seminar presentations while formative reflective techniques include class exercises, facilitation, and self-reflection guided by critical friends, supervisors, mentors, preceptors or peer observation. Other authors have invariable examined reflective journals, portfolios, logs, blogs, questionnaires, videos, and diaries ( Buckley *et al.* 2009; Miraglia and Asselin 2015). Important aspects of this techniques usually involve reflecting on daily practice or reflecting on goal oriented tasks. Despite their shortcomings in knowledge production, they appear to have an effect on changing attitudes, values, beliefs, and assumptions of individual participants increasing their sense of self-efficacy and purpose.

However, reflection has problematic areas of practice and research and issues that remain under investigation. The most important issue is the epistemological oxymoron since theory seems to contradict application. Until this is resolved or a consistent stance is adopted by researchers, educators and practitioners, this will be an important point of critique ( Ng *et al.* 2015). The next issue remains whether reflection truly enhances learning, self-understanding and improvement in practitioner skills or it simply has a positive effect on behaviour and mood. ( Mann *et al.* 2009; Prasko *et al.* 2012; Tsingos *et al.* 2015a)

## Self-study

Koster and van den Berg (2014) define self-study as the study of one’s own practice by the systematic exploration of what is happening, what participants think about their own practice, and what they want to change in their practice (p. 86). It has become popular in recent years and is in line with action research tradition followed by education. It is considered beneficiary for improving one’s own practice ( Loughran 2004; Pinnegar and Hamilton 2009d; Tidwell *et al.* 2009). This is the general definition followed but depending on the filed under investigation, self-study tends to relate to moral purpose, professional value and self-understanding within one’s own profession ( Lunenberg and Hamilton 2008; Lunenberg *et al.* 2010). Vicki Kubler LaBoskey phrased the concept of self-study and personal motivation quite eloquently in 2004 by saying:

*Our motivation in adopting a self-study stems also from the acknowledgement that we are as limited by our own personal histories and cultural identities as are our students, we cannot expand their horizons if we do not expand our own. Similarly, we cannot help them to detect and interrogate their biases if we do not detect and interrogate ours ( LaBoskey 2004: 840).*

It is clearly related to self-reflection with obvious similarities to theoretical underpinnings described in the previous section and its origins within the reflection scholarship ( Lunenberg *et al.* 2010), but it remains quite different from it. As Allard and Gallant (2012) describe it, self-study aims to systematize pedagogical reflection, but reflection meaning both as the motivation for self-study and as a means to be perform self-reflection and critical reflection as defined above ( Dinkelman 2003). If we think about it, this sort of reflection needed for self-study is something that will be subjected to scrutiny similar to that that we would scrutinize a research dataset of when reviewing a paper. This type of reflection is more strictly defined in a sense that because it will be studied and presented, it will need to be well defined and have rigor. As mentioned above, self-study is meant to be shared with your peers to improve your own practice. Hence, one quickly understands how self-study takes self-reflection from an abstract meaning to something more concrete meant to be analyzed and interpreted.

The dangers with self-study remain with issues of reliability. When studying one’s own self and practices, the teacher educator must be able to separate his personal feelings from the issue under investigation. Self-studies have been alleged to be frequently narcissistic and idiosyncratic lacking generazibility other qualitative research offers. The issue under investigation is part of his identity and in a sense defines the person. Hence, the teacher educator might come up with the issue of having to question his own actions or values that led him to them. In this context he needs to remain genuine and objective as possible acknowledging the subjective nature of the activity he is performing.

From the literature, a few methods have been used for self-study and are very closely related to reflective techniques. One important distinction is individual self-study and collaborative or group self-study; the second is considered to offer slightly more objectivity since more voices are participating and eventually heard, but some authors have argued that this is related to the goals of self-study and the professional or moral values underpinning the whole exercise ( Allard and Gallant 2012; Koster and van den Berg 2014). Invariably, biographies, core reflection, Socratic dialogue, and video-stimulated recall have been quite popular techniques, with the use of video been quite widespread over the last 20 years ( Gaudin and Chaliès 2015; Greenwalt 2008; Hamilton 2012). John Lyle defines video-stimulated recall “an introspection procedure in which (normally) videotaped passages of behaviour are replayed to individuals to stimulate recall of their concurrent cognitive activity” ( Lyle 2003: 861). Its implementation can vary significantly, with individual analysis or collaborative analysis and goal-purposed interpretation or reflexive dialogue (either alone or with peers) ( Samaras *et al.* 2016).

## Self-research

*My abhorrence of neoliberalism helps to explain my legitimate anger when I speak of the injustices to which the ragpickers among humanity are condemned. It also explains my total lack of interest in any pretension of impartiality, I am not impartial, or objective .. [this] does not prevent me from holding always a rigorously ethical position ( Freire 1998: 22).*

*Performance [auto]ethnography is the future of ethnography, and ethnography’s future is the seventh moment. In the seventh moment the dividing line between [auto]ethnography and ethnography disappears. The reflexive ethnographer becomes the guiding presence in the ethnographic text. In the seventh moment critical social science comes of age and becomes a force to be reckoned with in political and cultural arenas ( Denzin 2003: 259).*

The final step in the proposed model is how to analyze effectively the outcomes of self-study and self-reflection. The process of self-study is largely based on qualitative data and hence any analysis should be based on theory of qualitative research. Since many of the outcomes are related to following’ someone’s life (the teacher educators themselves), it needs to have an ethnographic narrative and because it relates to the self, autoethnography appears the most attractive ( Denzin 2003).

*Autoethnography is a subtype of ethnography in which an author uses self-reflection and writing to explore their personal experience and connect this autobiographical story to wider cultural, political, and social meanings and understandings ( Ellis 2004; Mills *et al.* 2010). The presence of autoethnography in research literature in education is strong. Maréchal (2010) defines autoethnography as “a form or method of research that involves self-observation and reflexive investigation in the context of ethnographic field work and writing” (p. 43) while Ellis (2004) defines it as “research, writing, story, and method that connect the autobiographical and personal to the cultural, social, and political” (p. xix). However, a consensus on the term’s definition is far from certain: autoethnography was initially described as insider ethnography, however a more inclusive definition is currently given by Adams *et al.* (2015):*

Autoethnography is a research method that uses a researcher’s personal experience to describe and critique cultural beliefs, practices, and experiences. Acknowledges and values a researcher’s relationships with others.. Shows people in the process of figuring out what to do, how to live, and the meaning of their struggles.. Social life is messy, uncertain, and emotional. If our desire to research social life, then we must embrace a research method that, to the best of its/our ability, acknowledges and accommodates mess and chaos, uncertainty and emotion ( Adams et al. 2015).

Traditionally five factors are used when assessing narrative papers that include analysis of both evaluative and constructive validity techniques. The criteria are given by Ellis (2004) and Richardson (2000):

• Substantive contribution. Does the piece contribute to our understanding of social life? ( Ellis 2004; Richardson 2000)
• Aesthetic merit. Does this piece succeed aesthetically? Is the text artistically shaped, satisfyingly complex, and not boring? ( Ellis 2004; Richardson 2000)
• Reflexivity. How did the author come to write this text? How has the author’s subjectivity been both a producer and a product of this text? ( Ellis 2004; Richardson 2000)
• Impactfullness. Does this affect me emotionally and/or intellectually? Does it generate new questions or move me to action? ( Ellis 2004; Richardson 2000)
• Expresses a reality. Does this text embody a fleshed out sense of lived experience? ( Ellis 2004; Richardson 2000)

However a proper theoretical stance is needed: the teacher educator needs to define whether he will follow phenomenology, grounded theory or another interpretive stance such a reflexivity, narrative inquiry or critical pedagogies. Considering that the objects of analysis are written texts or audio-visual materials, an issue of coding comes into place as well ( Adams *et al.* 2015; Denzin 2003; Ellis 2004).

Springer’s Book Series Self-Study of Teaching and Teacher Education Practices offers an insightful series of volumes which explore the research nature of self-study and how to proceed. Self-study is considered as a genre of qualitative research and as such it should be researched under that umbrella. Trustworthiness and being trustworthy during practice, data collection and data interpretation is necessary. Next triangulation with other sources is needed to establish the strength of a result. Finally the need for pragmatic but also theoretic conclusions is stressed ( Pinnegar and Hamilton 2009a, b, c).

An interest aspect is given in another book of this series Research Methods for the Self-study of Practice ( Fitzgerald *et al.* 2009). Since self-study is happening in a collaborative manner, co/autoethnography is becoming prevalent, in a sense that interpersonal relationships, cultural beliefs and more abstract principles that relate with the interplay of many people cooperating together on a certain research issue, affect the outcome of the analysis ( Coia and Taylor 2009).

## Epilogue and Discussion

**Figure 1.**
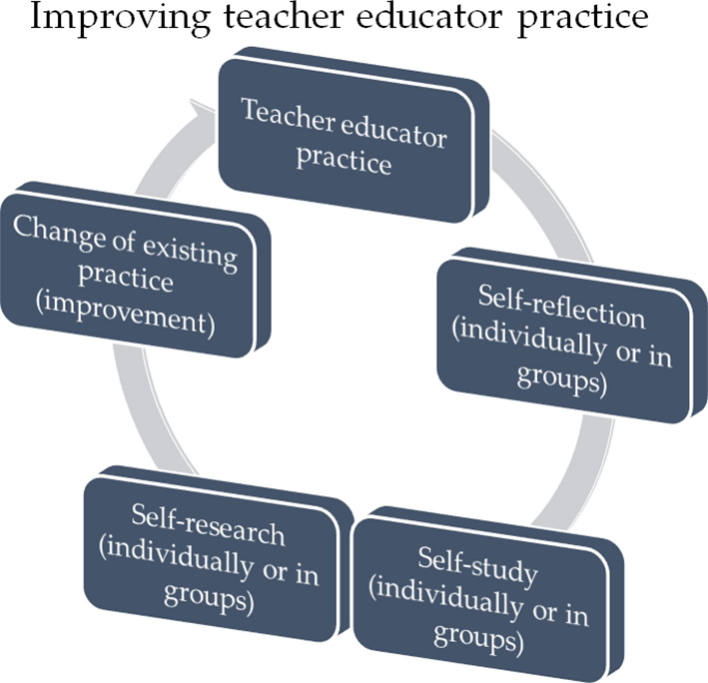
Chain of improvement of teacher educator practice

In the present essay it has been advocated that a chain of self-reflection, followed by self-study and eventually by self-research may lead to improvement of teacher educator practice. This continuous cycle is depicted in Figure 1. Some general comments that I think are quite important in achieving the improvement of teacher educator practice are given below.

The teacher educator needs to understand his identities and question them. Each identity is associated with various other concepts, which could be values, professionalism, personal beliefs, cultural beliefs and so on, an all this comes into effect when your identity is investigated. Hence, self-understanding of one’s identity is imperative for this kind of improvement. The self-critiquing stance that in essence applies subjectivity and constructivism to your beliefs is more close to critical pedagogical approaches compared to behaviorist positivist approaches in education.

The next thing the teacher educator should understand is reflection. There are many types of reflection but reflection remains the cornerstone of improvement. However, reflection should be deep and not superficial, coming about as a mere dictation from a regulation body that suggests this to ensure professional registration. This reflection requires a reflexive stance where improvement is your aim through constant inquiry and also questioning of your existing beliefs. The quotation by Paulo Freire (1998) presented in the Self-research section and the article by qualitative researcher Norman Denzin (2003) are enlightening towards this path of inquiry. Reflection is currently suffering from its own paradoxes in that although it could belong (and maybe should) to critical pedagogical approaches, it is being adapted (and adopted) by utilitarian reductionist practitioners, possibly eschewing it from its original conceptions by Donald Schön, John Dewey and Jack Mezirow.

Next important step is familiarization with self-study. Self-study is largely based on reflection theory and techniques but it something new altogether. In my eyes, it is the introduction of a new field, that proposes that systematic analysis of one’s own practice can lead to improvement of his own practice. Issues that come up and are related to self-research and need to questioned and clarified are data collection techniques, data analysis and ensuring trustworthiness (reliability). Research should be ethical, non-narcissistic and non-idiosyncratic. However, this self-improvement stance could also be challenged as a difficult one because it essentially expects anyone who realizes his/her identity as a teacher educator to become literally a researcher. However, if one excludes the final step of self-research/self-study, personal practice can be improved through various techniques of self-reflection. Another point to contest is the possibility of building-up the teacher educator ego since their own practice becomes their mode of operation, depriving them from a perspective on others’ views.

That previous comment brings highlights the importance of individual versus collaborative self-reflection/self-study/self-research. Collaborative self-reflection along with the impact it has on study and research, can deal with the some of the shortcomings posed by individual self-study. Narcissism and idiosyncrasy tend to become obsolete when working in the presence of a team.

In conclusion, this present essay has suggested a model of improving teacher educator practices by acknowledging the multiple identities a teacher assumes in daily practice. This requires a self-critiquing stance of personal actions and the development of new skills in teaching practice.

## Notes On Contributors

Konstantinos C. Fragkos is with UCL Medical School and University College London Hospital. He finished his studies at the National University of Athens, Greece and is a qualified physician. His research interests include systematic reviews, meta-analysis, education, reflective practice, and clinical nutrition with several publications in these areas.
