# Supplementary material for: Exploring whether (and how) self-reflection can improve practice as a teacher educator
Source: MedEdPublish (2016). 2018 Mar 23;7:67. [Version 1] doi: 10.15694/mep.2018.0000067.1 (PMC10711950; doi:10.15694/mep.2018.0000067.1)
Supplement: Peer review history

# Reviewer report: Reviewer response for version 1

**DOI:** 10.21956/mep.19537.r28933

**Published:** 2019-02-23

**Authors:** Trevor Gibbs

**Affiliations:** 1 AMEE

This review has been migrated. The reviewer awarded 4 stars out of 5 An interesting paper to read and one that provides a very useful reference list. I would have liked to see the author complete the Take Home Messages section because I feel that it would focus a little more of what the main messages were.The paper gave support to the need to develop the educator and that they are not just borneI would recommend this paper to all those about to embark upon any aspect of medical education.

Reviewer Expertise:

NA

No decision status is available

# Reviewer report: Reviewer response for version 1

**DOI:** 10.21956/mep.19537.r28931

**Published:** 2018-04-08

**Authors:** Stewart Hazelton

**Affiliations:** 1 Bond University

This review has been migrated. The reviewer awarded 3 stars out of 5 Strengths of PaperThe paper chosen gave a very broad analysis of the components and theory behind self-reflection by educators to improve their craft. The author provided a comprehensive deconstruction of self-reflection through literary review. Poignant acknowledgements of having a balance between foundations in theory and real-world experience underpinned the direction of the article. Moreover recognises inherent biases in the self-reflection profess that may impede an appropriate self-evaluation. The graphic representation of the overarching points disgusted provided an easily consumable method of tying salient points components together. Weakness of PaperThe paper is highly technical and philosophical. This was ironic to me, as self-reflection is a very practical ‘everyday skill’, that’s carried out in seconds, hours, or days, in order to self-correct and improve. The language and technical approach employed perhaps detracted away from its useability to the layperson. Confining it’s understanding to those who academically specialise in this area. Thus this very practical and vital skill was analyses and philosophised to a level of impracticality to everyday thought. A suggested improvement for a revised edition could be to include a section that translates this highly theoretical and philosophical paper into a practical methodology that can be employed by the everyday clinician. Flavour of Interest (***)Given the above, I would imagine this paper is more appropriately directed towards the educator specialising in the ‘theory of self-reflection’. Key points to then be distilled and disseminated to those who will use this everyday method of self-correction. For students or clinicians that primarily teach by the bedside with incidental student encounters, this article is probably too theory heavy.

Reviewer Expertise:

NA

No decision status is available

# Reviewer report: Reviewer response for version 1

**DOI:** 10.21956/mep.19537.r28932

**Published:** 2018-04-05

**Authors:** Anthony David.M

**Affiliations:** 1 Soho

This review has been migrated. The reviewer awarded 4 stars out of 5 An enjoyable read. The concept of Reflection aiding one's development as a Medical Educator is very valid. The cycle proposed is a do-able one and which many of us can follow. This concept I feel is very useful for some uf us who double up as Teacher Educators taking active part in Faculty Development programs.

Reviewer Expertise:

NA

No decision status is available

# Reviewer report: Reviewer response for version 1

**DOI:** 10.21956/mep.19537.r28930

**Published:** 2018-03-23

**Authors:** Subha Ramani

**Affiliations:** 1 Harvard Medical School, Brigham and Women's Hospital

This review has been migrated. The reviewer awarded 5 stars out of 5 I thoroughly enjoyed reading this essay and agree with all the aspects of 'self' in this model for medical teachers. It is important for teachers to reflect on their actions, do a self-study of effective and ineffective strategies and develop commitment to change. I also believe the recommendations and proposed model will help teachers in this journey of self-exploration.There are some challenges to this process that must be discussed. First, the world of medical education has assumed that all graduates will just begin to teach spontaneously. It is gratifying that more and more teachers are stimulated to participate in staff development activities. Second, clinicians who teach face increasingly full professional plates (platters) and where is the time to reflect? If educational leaders believe that this voyage of self is important, they must establish explicit expectations and provide time and space to reflect. Lastly, there are research reports that indicate that professionals do not always accurately calibrate their strengths and weaknesses. This might initiate an erroneous chain of self-reflection. Perhaps, peer observation and facilitated reflection can be of enormous value.All those who teach in health professions education will find this paper to be of value in guiding how they approach their teaching.In Ronald Harden's 3-circle Dundee model, reflection can be placed at the highest circle- "the right person doing it".Most enjoyable and I will be using this essay to continue to grow as a teacher.

Reviewer Expertise:

NA

No decision status is available
